# Evaluating the Effect of Activity and Environment on Fall Risk in a Paradigm-Depending Laboratory Setting: Protocol for an Experimental Pilot Study

**DOI:** 10.2196/46930

**Published:** 2023-05-10

**Authors:** Kim Sarah Sczuka, Marc Schneider, Michael Schellenbach, Ngaire Kerse, Clemens Becker, Jochen Klenk

**Affiliations:** 1 Department of Clinical Gerontology Robert-Bosch-Hospital Stuttgart Germany; 2 Institute of Computer Science University of Applied Science Ruhr West Mülheim an der Ruhr Germany; 3 Department of General Practice and Primary Health Care University of Auckland Auckland New Zealand; 4 Digital Geriatric Medicine Heidelberg University Heidelberg Germany; 5 Institute of Epidemiology and Medical Biometry Ulm University Ulm Germany; 6 IB University of Health and Social Sciences Study Center Stuttgart Stuttgart Germany

**Keywords:** fall risk, fall risk factor, fall-related activity, laboratory setting, study protocol, fall, fall risk model, older people, elderly, analysis of fall

## Abstract

**Background:**

Knowledge about the causal factors leading to falls is still limited, and fall prevention interventions urgently need to be more effective to limit the otherwise increasing burden caused by falls in older people. To identify individual fall risk, it is important to understand the complex interplay of fall-related factors. Although fall events are common, they are seldom observed, and fall reports are often biased. Due to the rapid development of wearable inertial sensors, an objective approach to capture fall events and the corresponding circumstances is provided.

**Objective:**

The aim of this work is to operationalize a prototypical dynamic fall risk model regarding 4 ecologically valid real-world scenarios (opening a door, slipping, tripping, and usage of public transportation). We hypothesize that individual fall risk is associated with an interplay of intrinsic risk factors, activity, and environmental factors that can be estimated by using data measured within a laboratory simulation setting.

**Methods:**

We will recruit 30 community-dwelling people aged 60 years or older. To identify several fall-related intrinsic fall risk factors, appropriate clinical assessments will be selected. The experimental setup is adaptable so that the level of fall risk for each activity and each environmental factor is adjustable. By different levels of difficulty, the effect on the risk of falling will be investigated. An 8-camera motion tracking system will be used to record absolute body motions and limits of stability. All laboratory experiments will also be recorded by inertial sensors (L5, dominant leg) and video camera. Logistic regression analyses will be used to model the association between risk factors and falls. Continuous fall risk will be modeled by generalized linear regression models using margin of stability as outcome parameter.

**Results:**

The results of this project will prove the concept and establish methods to further use the dynamic fall risk model. Recruitment and measurement initially began in October 2020 but were halted because of the COVID-19 pandemic. Recruitment and measurements recommenced in October 2022, and by February 2023, a total of 25 of the planned 30 subjects have been measured.

**Conclusions:**

In the field of fall prevention, a more precise fall risk model will have a significant impact on research leading to more effective prevention approaches. Given the described burden related to falls and the high prevalence, considerable improvements in fall prevention will have a significant impact on individual quality of life and also on society in general by reducing institutionalization and health care costs. The setup will enable the analysis of fall events and their circumstances ecologically valid in a laboratory setting and thereby will provide important information to estimate the individual instantaneous fall risk.

**International Registered Report Identifier (IRRID):**

DERR1-10.2196/46930

## Introduction

Falls are the most prominent cause of death due to injuries in older persons [1]. Falls are also a common event leading to serious physical consequences such as fractures as well as reduced quality of life, loss of independence, and institutionalization in nursing homes. In total, 1 in 3 community-dwelling people aged 65 years or older fall at least once a year, and half of them fall multiple times [2,3]. Falls are one of the leading health conditions associated with disability in populations aged 60 years and older [4]. Therefore, research on falls and fall-related aspects has been intensified in the past years.

The current Cochrane Review on fall prevention in community-dwelling older people lists several effective exercise programs [5]. However, there is limited impact at the population level with the risk of falls being reduced between 13% for balance and functional exercises and 22% for multiple types of exercises including resistance training. Tai Chi reduced the fall risk by 20%. Even less effective are interventions in residential aged care [6], where the effect of exercise on risk of falling makes little or even no difference. These numbers clearly show the need for further improvements in fall prevention programs. The limited effectiveness might be due to lacking task-specific exercises required to prevent falls. Perturbation training is a task-specific intervention with the aim to improve a person’s reactive balance control under safe laboratory conditions [7]. Recent perturbation studies showed a significant reduction of falls among healthy older adults as well as patients with Parkinson disease and stroke [8]. Furthermore, clinically relevant reductions of fall incidence were shown in frail older adults. It appears that treadmill-based approaches, which enable multiple types and directions of perturbations, might be the most practical method and might be of the most benefit. Although recent studies showed the feasibility of perturbation-based training, conclusions related to the optimal perturbation number and type are difficult to make. Another promising task-specific approach is gait adaptability training [9]. During gait adaptability training, participants respond to environmental challenges by making quick and appropriate voluntary adjustments of gait patterns. A systematic review and meta-analysis by Nørgaard et al [9] showed a reduction of 42% in fall rate, and the proportion of fallers could be reduced by 43% in older adults. These results are in accordance with the results of perturbation-based interventions. It is likely that even more efficient strategies could be informed by a more comprehensive understanding of the causal pathways of falling and fall risk.

Analyses of fall risk factors suggest that we do not fully understand their complex interplay and factors triggering fall events. Meta-analyses identified about 30 fall risk factors for falls in community-dwelling older adults [10]. These include fall history, balance, and gait problems. The published fall risk models are unsatisfying considering the externally validated predictive accuracy. A systematic evaluation of 4 fall prediction tools in residential care showed that each model was unsuitable due to poor precision [11]. Sensitivity and specificity ranged from 0.50 to 0.80 and 0.32 to 0.80, respectively. The web-based FRAT-up (Fall Risk Assessment Tool) fall prediction tool, developed for community-dwelling older people, has an area under the curve of 0.65 and showed heterogeneity in results from different cohorts [12]. The performance of these fall risk models in individuals has been moderate at best and is therefore not sufficient to guide clinical practice and health policy.

With the rapid development of eHealth devices including body-worn sensor technology, small wearable and unobtrusive devices are now available; this can provide objective measures of physical activity, kinematics of human movement [13], and falls during everyday life [14]. Additionally, it is possible to examine environmental factors such as stairs, weather conditions, or indoor or outdoor environments using combinations of monitoring systems. Adding these measurements such as gait intensity and gait complexity to clinical risk factors improves the area under the curve for fall prediction from 0.68 to 0.82 [15]. However, the most important risk factor still remains the history of a prior fall. Other recent studies using information and computer technology to predict falls showed similar results. Internal and external validity is not yet firmly established as some models have been developed and validated on the same samples; thus, replication is required [16,17]. Furthermore, most of the above information and computer technology studies have limitations. They have used fall history, clinical fall risk assessment, or both to define fall risk, instead of the gold standard measure, which remains the prospective fall ascertainment [18].

Even if it is possible to replicate the results, there is still a large number of falls that are not predicted. Furthermore, a rarely discussed aspect in fall risk estimation is the time frame of prediction in which the event of interest occurs. A simple distinction between fallers and nonfallers is insufficient as most people will fall if the period of observation is long enough. The time window of prediction in most studies is 1 year based on the follow-up duration of the prospective fall recording. However, the question remains whether this follow-up period is sufficient or which period is necessary to cover a relevant fall risk. This question is also relevant in terms of feasibility for a clinical trial. Furthermore, the necessary duration may also depend on whether a fall is more likely due to a chronic disease or if a fall is merely due to random unpredictable circumstances. For effective and acceptable fall prevention measures on an individual level, much shorter prediction windows may be needed. In this context, an intervention that is able to directly test fall risk by exposing participant to hazardous ecologically valid situations would be conceivable.

Rubenstein and Josephson [19] drafted a conceptual model, which includes intrinsic risk factors, extrinsic risk factors, and precipitating causes. Klenk et al [20] further developed this concept by structuring the components and adding the dynamic nature of fall risk and thereby suggested a modified model, which includes intrinsic risk factors and the exposure. The model considers that fall risk factors may change over time due to individual and temporary exposure to hazardous situations, environmental factors, and unexpected events. Furthermore, different activities pose different levels of hazard, for example, it is riskier to run than to walk.

The complex interplay of different factors results in an individual instantaneous fall risk. If a person with impaired balance walks over slippery ground, the risk of falling is increased compared to a person with balance impairment who walks fast on dry ground.

The impact of one or more activities undertaken at the same time varies depending on cognitive capacity and environmental conditions. For instance, walking on an uneven surface while talking may result in a fall, whereas talking and walking on a smooth surface may not. Similarly, an unexpected event, such as a perturbation, or a visual startle may disrupt an otherwise simple maneuver. We posit that all mentioned components of risk do not simply sum linearly to a total, but the way they could be combined is likely to be complex. Components change over time, and the impact of the components could be additive, multiplicative, or weighted. A fall event occurs when the interplay of intrinsic risk factors and exposures reaches a threshold.

To operationalize the different components of the dynamic fall risk model, real-world fall events with detailed information about the fall events are necessary. Although falls are common, it is challenging to capture real-world fall signals due to the limited recording capacities of sensor devices. However, the Fall Repository for the Design of Smart and Self-adaptive Environments Prolonging Independent Living (FARSEEING) consortium, funded by the 7th EU Framework Program for Research, started to collect an ample data set of real-world falls by a preplanned collaboration of many research groups. Within the European FARSEEING project, a database of more than 200 validated real-world falls of older people measured by body-worn sensor technology was compiled [21]. First results in the field of fall detection show the potential to bridge existing knowledge gaps [22,23]. The fall events, collected in the FARSEEING database, provide the possibility to obtain knowledge concerning ecologically valid real-world fall scenarios, activities that were performed immediately before the fall as well as environmental factors.

However, most sensor devices are not able to provide information about context and environmental conditions as well as intrinsic fall risk factors triggering a fall event. The information available so far is not sufficient. A promising approach to add missing information is the re-enactment of known fall events. Connell and Wolf [24] previously proposed the method to validate subjective fall reports. Participants were interviewed and asked to re-enact in detail (if they felt comfortable) all activities, body movements, body part placements, and interactions with the environment at the location of the incident to obtain more precise information. Re-enactment is suitable in order to improve simulation protocols and produce more realistic fall simulations [25]. However, to further improve simulation settings and gain more knowledge, controllable and repeatable conditions are needed, which also realistically correspond to everyday situations. For certain everyday situations, these criteria can be met within a laboratory setting. This approach allows intrinsic risk factors as well as instantaneous dynamic risks (eg, activities and environmental conditions) and their complex interplay to be investigated in a controlled condition in realistically re-enacted everyday situations and thereby to provide a more comprehensive understanding of the causal mechanisms of falling and fall risk.

Therefore, we developed a setup to provide an experimental environment to simulate and measure selected paradigms under different conditions and adjustable factors in order to find individual fall thresholds. In this paper, we describe an experimental laboratory setting based on 4 ecologically valid real-world scenarios and the planned pilot study that will provide the data to operationalize a first simplified dynamic fall risk model.

## Methods

### Study Design

This study has an experimental design to analyze the interplay between intrinsic fall risk factors and exposure to hazardous environmental conditions and activity. All participants will obtain extensive clinical and quantitative assessment as well as perform the study protocol to evaluate the effect of different conditions on the individual fall risk in accordance with the study protocol at the Robert-Bosch-Hospital, Stuttgart.

### Eligibility Criteria

German-speaking, community-dwelling, healthy as well as prefrail people aged 60 years or older will be recruited. Exclusion criteria are current diagnosis of a serious neurological or sensory disease; uncorrected visual difficulties; dizziness; uncontrolled or serious cardiovascular, metabolic, or psychiatric disorders; cognitive restriction and manifest daily relevant dementia; contraindications for perturbations, including osteoporosis, joint replacement, joint stiffening, severe spinal disease, or recent fracture as well as people who are unable to walk at least 5 minutes freely.

### Recruitment

Participants (N=30) will be recruited from an existing pool of subjects who have already participated in studies and have given their written consent to participate in further studies. To obtain sufficiently meaningful results, see if the experimental setup needs adaption, and test the feasibility within this pilot study, sample size is sufficiently high and chosen pragmatically but not estimated [26]. Overall, 20 subjects will be used for the development of the prototypical fall risk model, and 10 subjects will be used for validation. Ideally, half of the subjects would be considered prefrail, but prefrail subjects may drop out during the experiment or will not participate at all. Participants will be contacted via telephone and informed about the study and the procedure if they are interested.

### Clinical Assessments

To identify several fall-related intrinsic fall risk factors relevant to the model development and describe the cohort, appropriate clinical assessments were selected. The estimated total duration of clinical assessments and the trial protocol will be 120 minutes. Clinical assessments will be carried out for all participants before the experimental protocol. All assessments will be performed by trained assessors.

Clinical data like sex, age, body weight (BW), physical activity as well as medical history will be assessed. Furthermore, general state of health and comorbidity will be assessed using the Functional Comorbidity Index [27].

Occurrence of falls during the last 12 months will be inquired. For the assessment of fall-related self-efficacy, the Short Form of the Falls Efficacy Scale-International will be used. This scale has been shown to be highly related to previous and subsequent falling [28].

Physical performance will be assessed by the Short Physical Performance Battery [29] and the Timed Up-and-Go Test [30]. People who need >12 seconds for the Timed Up-and-Go test will be classified as individuals at a higher risk of falling [31]. Regarding the cutoff value of 12 seconds, we followed Jansen et al [31], who lowered the threshold of 13.5 seconds originally defined by Barry et al [32]. Additional gait analysis will be conducted for habitual and fast walking speed. Furthermore, gait analysis will be performed under dual-task conditions (fast gait speed and counting backward minus 7 beginning with 100). Cognitive condition will be examined using the Trail-Making-Test [33]. Assessment measurements are listed in Textbox 1.

**Listing of demographic and medical data as well as assessments.**

**Sociodemography**

- Age and gender

**Medical information**

- Height, weight, BMI, comorbidities, occurrence of fatigue, and weight loss

**Physical activity status**

- Questionnaire: physical activity during the last 2 weeks
- Fall history and fall-related injuries in the past 12 months

**Fear of falling**

- Short Falls Efficacy Scale International [28]

**Motor function**

- Short Physical Performance Battery [29]
- Timed Up-and-Go [30]
- Gait performance (usual or fast pace, dual-task condition)

**Cognitive status**

- Trail-Making-Test [33]

### Experimental Setup

#### Overview

All participants will perform the same protocol. The fall simulation experiments will collect the data needed to develop the simplified mathematical dynamic fall risk model. Ideally, the activities performed should not give the impression of a simulation for the subjects. They should behave as they would naturally, and our objective is to provoke a reaction that ideally cannot be consciously influenced by the individual. Due to safety reasons and to avoid injuries, participants will wear a safety harness that prevents any contact of the body with the ground except the feet. The safety system is adjusted accordingly. Also, the treadmill can be stopped immediately at any time by the trained study assistant who is always standing next to the treadmill at an emergency stop button during the measurements. The test protocol will comprise a series of the selected activities without and in combination with mediating environmental factors. The experimental setup has to be adaptable meaning that it is possible to adjust the level of fall risk for each activity and each environmental factor. The level of difficulty will be stepwise increased for each participant. The registration of more than 30% of the BW on a load cell attached to the safety harness will be used to define a fall event [34]. Several prototypical examples of everyday fall scenarios have been recorded in the FARSEEING database [21]. Based on this data, we will consider four prototypical scenarios, which are common and relevant from a public health perspective: (1) falling during walking backward while opening the door, (2) falling due to slippery surface, (3) falling due to a trip over an obstacle, and (4) falling in a bus while the bus is starting.

**Figure 1:**
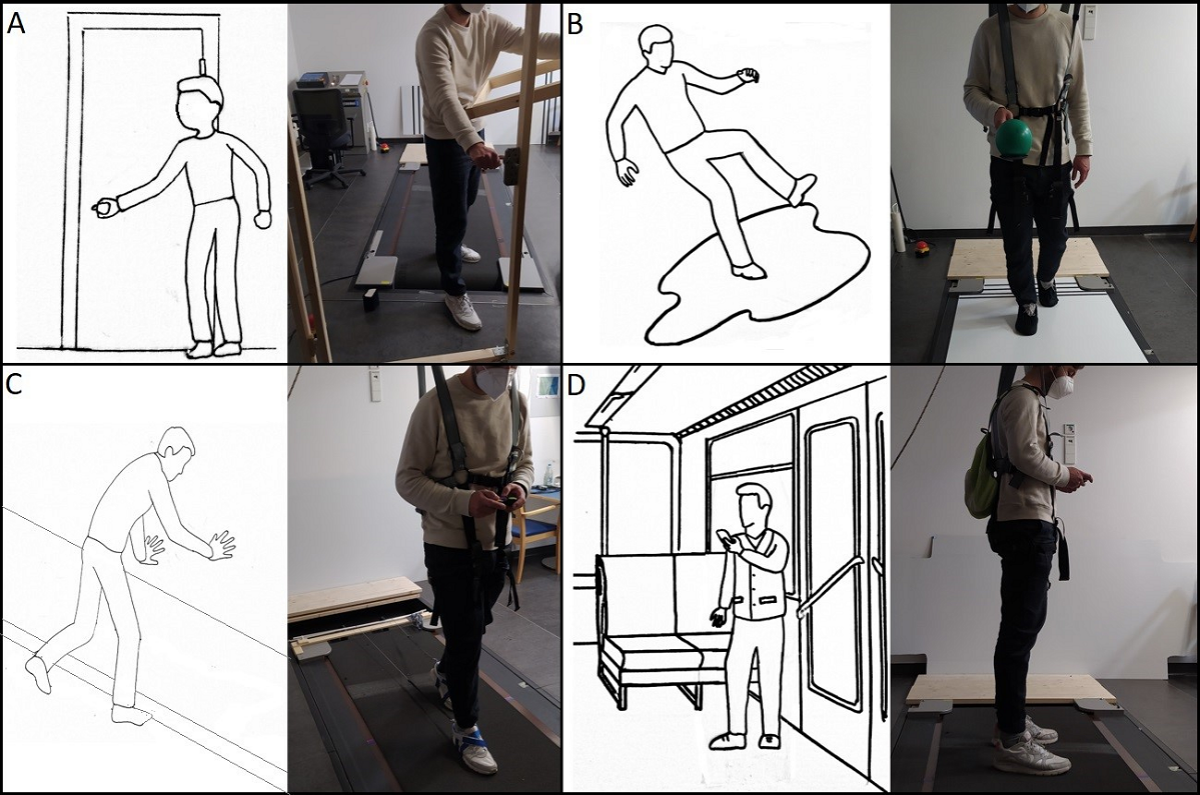
Illustration of the 4 selected everyday fall-related scenarios and their implementation in the laboratory. (A) Opening a door, (B) slippery surface, (C) tripping over an obstacle, and (D) usage of public transportation.

An experimental setup for each selected scenario will be established in the laboratory. The 4 selected fall scenarios and their implementation in the laboratory are shown in Figure 1.

#### Opening a Door

**Figure 2:**
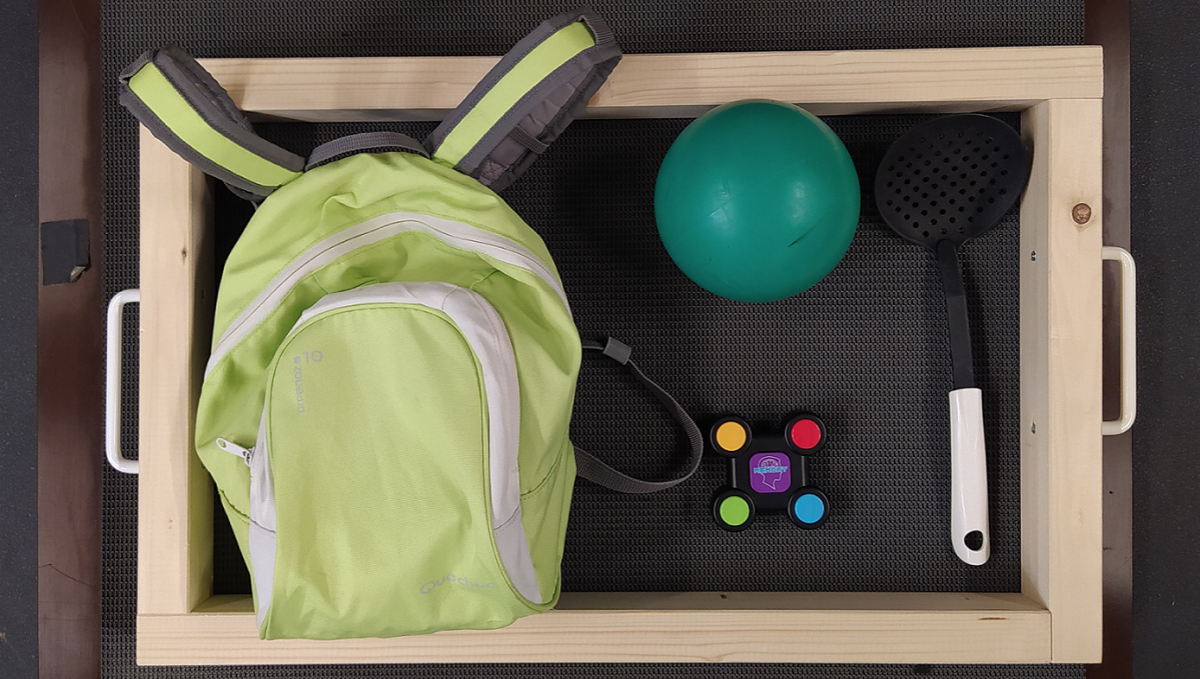
Equipment for additional tasks: carrying a wooden frame that represents a laundry basket, carrying a backpack, balancing a ball on a scoop, and playing with a memory buzzer.

In front of the participant is a custom-built wooden door–like frame. Participants will be instructed to open the door. Following this baseline measurement, the participants will be repeatedly asked to open the door with additional activities and environmental factors: opening the door as fast as possible, carrying a laundry basket, balancing a ball on a scoop, and opening a rough-running door. The laundry basket will be represented by a wooden frame with handles to cause less occlusion (Figure 2). To simulate a rough running door, the door will be equipped with a neodymium magnet that increases the resistance when opening.

#### Slippery Surface

Within this scenario, participants will wear shoe covers while walking over an aluminum di-bond plate, so that there will be reduced ground friction. To make it harder to maintain balance, the following factors will be stepwise added and combined: walking as fast as possible, balancing a ball on a scoop, usage of a memory buzzer to increase cognitive load (Figure 2), walking up and down the inclined (5%) treadmill (h/p/cosmos venus; length 200 cm, width 75 cm, h/p/cosmos sports medical GmbH).

#### Tripping Over an Obstacle

Two retaining plates will be attached to the participants’ heels with a custom-built fixation. At first, the participants will walk on the treadmill with self-selected gait speed in order to get used to the treadmill. After habituation, a neodymium magnet will be stuck to the retaining plate at the participants’ heels (magnetic adhesive force=41 kg). The magnet will be connected to inelastic cords and will ensure that the blockage of the leg can be broken by detaching the magnet when swinging the affected leg forward. The 2 inelastic cords will be attached behind the treadmill, which will be shortened or lengthened with low resistance by a coil spring while the participant is walking. The continuous length variation of the cords can be interrupted at the push of a button. When pushing the button at the end of the stance phase, the subsequent swing phase is perturbed. In random time intervals, the participants’ feet will be blocked for a very short moment until the button is released or the magnet is detached from the retaining plate in order to perturb the swing phase but enable continuous walking afterward without stopping or limping. The series of perturbations will be determined in advance and will be unknown to the participants. Additional factors will be carrying a laundry basket, playing with the memory buzzer, and increasing the self-selected gait speed by 33%.

#### Usage of Public Transportation

To simulate the starting of a bus, participants will stand on the resting treadmill, and suddenly the treadmill belt will be accelerated. By means of the coscom interface protocol coscom v4, acceleration values and duration as well as target speed can be accessed. A custom application is written in Python for this purpose. Each participant will start with several forward and medio-lateral right perturbations with lower intensity to become familiar with the perturbations and choose an individual standing position that is not allowed to be changed during the experiments. The selected position shall correspond to the position that the participants would also choose on a bus. Concerning forward perturbations, the treadmill will be accelerated for 1 second and thereby will reach a maximum acceleration of 1.61 m/s^2^. Participants will stand against the running direction of the treadmill belt. Concerning sideward perturbations, the treadmill will accelerate for 0.75 seconds and will reach a maximum acceleration of 1.51 m/s^2^. To realize sideward perturbations, the participants will stand crosswise to the running direction of the treadmill belt. In Stuttgart, Germany, Lindemann et al [35] measured the incident-type acceleration from standing extreme values of acceleration of 3.37 m/s^2^ in buses. Therefore, the maximal possible acceleration values within this study will be lower compared to the real-life ones, but due to safety conditions and technical limitations, no higher acceleration will be realizable. Acceleration from resting state and stopping to standing of the treadmill belt depends on length of the treadmill. The entire acceleration and deceleration process has to result in a movement of the treadmill belt less than 2 m. By doing so, the subjects will not fall off the treadmill in case they would not do any recovery steps. After baseline measurement, which represents the participants’ reaction to the perturbation without an additional factor, one or more additional factors will be combined. The additional factors for this fall scenario will be hearing bus-related noise, wearing a backpack, and playing with the memory buzzer. The backpack will be loaded with 10% of the subject’s BW and will be carried symmetrically. Sahli et al [36] demonstrated that carrying a backpack loaded with 10% BW seems to alter balance of adolescents with mild idiopathic scoliosis.

#### Measurement Instruments

An 8-camera VICON T10 system (Vicon Motion Systems Ltd UK) will be used to record absolute body motions and limits of stability using the Plug-in-Gait full-body marker model. The VICON motion tracking system is able to measure a person’s movement and duration very precisely.

To record further parameters, we will use 2 wearable sensors (Blue Trident inertial measurement unit, Vicon Motion Systems Ltd UK) with a combined triaxial accelerometer (sampling rate 1125 Hz; range ±16 g) and triaxial gyroscope (frequency 1125 Hz; range ±2000°/s). The sensors will be fixed with a custom-built elastic belt. Two synchronized Blue Trident inertial measurement units (Vicon Motion Systems Ltd UK) will be connected to the system. One will be attached to the lower back at L5 position, and the other one will be attached to the dominant leg at ankle height. To validate the results from the body-worn sensors, all laboratory experiments in this project will also be recorded by a video camera (Basler pilot piA640-210gc).

### Participants’ Safety and Adverse Events

All study participants will receive instructions for safe performance (shoes and safety harness) and will be asked to perform all paradigms as long as they feel comfortable. During the laboratory experiments, the participants will be protected from falling by a safety harness. Serious adverse events related or potentially related to the study participation will be reported to the responsible ethic review board.

### Ethics Approval

The study will be conducted according to Good Clinical Practice and in accordance with the principles of the Declaration of Helsinki 1964 and all subsequent revisions. All procedures are approved by the local ethics committee (University of Tübingen, 245/2018BO2). All participants will have to give their written informed consent and will be insured. All project staff is bound by confidentiality. The results of the data collection in written form are subsequently digitized for evaluation. All data are pseudonymized. If a subject withdraws his or her consent, the corresponding data will be deleted immediately.

### Outcome Measure and Data Analysis

Primary outcome will be the number of falls experienced by the participants during the experiment. Furthermore, the concept of margin of stability (MoS) will be used as a continuous measure to quantify the risk of falling [37]. MoS indicates the distance between the base of support of a person and the extrapolated center of mass in static and dynamic conditions.

As previously described, sociodemographic and medical information, physical activity status, fear of falling as well as motor function will be assessed and subsequently included as factors in the dynamic fall risk model.

The estimation of a person’s individual fall risk will be based on the collected data. In the first step, logistic regression analyses will be used to model the association among activity characteristics, environmental fall risk factors, and falls. The optimal model for each scenario will be selected by receiver operating characteristic analyses. In the second step, continuous fall risk will be modeled by generalized linear regression models using MoS as outcome parameter. Nonlinear associations and interactions will be tested and modeled, if necessary.

Participants who will drop out or incomplete data will be included as long as the data set for at least 1 scenario is complete. It is planned to use the data sets of 20 subjects for model development, the other 10 data sets will be used for validation.

## Results

This study was initially designed to begin recruitment in July 2020 after ethics approval has been received. First, measurements took place in October 2020, but not all subjects could be measured as planned because of the COVID-19 pandemic. Due to access restrictions in hospitals and difficulties in recruitment as a result of a lack of willingness in the target group to participate in the study, further measurements were not conducted until October 2022. In the meantime, the already recorded data were processed and prepared for subsequent analyses. Up to February 2023, a total of 25 of the planned 30 subjects have been measured. The submission of the manuscript including complete results is expected by August 2023.

## Discussion

### Principal Findings

Older people fall frequently with serious consequences, including injury and hospitalization. However, the knowledge about the causal factors leading to falls is still limited, and fall prevention interventions need to be more effective to limit the otherwise increasing burden caused by falls in older people. Therefore, the aim of this project is to operationalize a dynamic fall risk model by focusing on the time period close to the fall event where all domains of the dynamic fall risk model cumulate. This will help to further understand the complex interplay triggering a fall event.

To the best of our knowledge, this planned study is the first approach to assess the complex interplay of intrinsic risk factors and environment and activity exposure under controlled but realistic experimental conditions. We will consider 4 prototypical scenarios that are common and relevant from a public health perspective. The model-based assessment of fall risk under (simulated) real-life conditions in a safe environment will enable the systematic analysis of the complex relationship between fall risk factors. Based on the planned analysis of the MoS, even small perturbations that will not immediately lead to a fall show an effect on a person’s dynamic stability. Such an in-laboratory fall simulation environment might also serve as an outcome parameter for intervention studies. A rarely discussed aspect in fall risk estimation is the required time window of prediction in which the fall event occurs. A simple distinction between fallers and nonfallers is not sufficient as most people will fall if the period of observation is long enough. The time frame of prediction in most studies is 1 year. However, the question remains whether this follow-up period is sufficient or which period is necessary to cover a relevant fall risk. For effective and acceptable fall prevention measures on an individual level, much shorter prediction windows may be needed. This would reduce costs and speed up the development of new fall prevention measures. In this context, an intervention that is able to directly test fall risk by exposing participant to hazardous ecologically valid situations would be conceivable.

### Limitations

Nevertheless, this study has some limitations. First, the sample size will be relatively small. However, the subjects will be tested extensively with a standardized protocol. This setting will allow the extraction of relevant information and the development of the dynamic fall risk model. Although the laboratory setting will approach natural conditions, it will remain artificial insofar that subjects will partially walk on a treadmill in a sparse environment. Furthermore, the subjects will be aware of the objective of the study: reaching the fall threshold by creating a situation that becomes stepwise more challenging. In contrast, falls are a sudden and mostly unexpected event during everyday life, and normally, a person would not anticipate a falling situation. Another aspect is the acceleration of the treadmill belt. To simulate the starting of a bus, the treadmill will accelerate with a maximum of 1.61 m/s^2^ due to safety reasons and technical limitation. Lindemann et al [35] measured extreme values of acceleration of 3.37 m/s^2^ in buses. Therefore, the maximal possible acceleration is lower compared to the real-life ones. However, we believe that this setup will allow analysis of the association of environmental as well as intrinsic factors and activity exposure and thereby will lead to a better understanding of the complex interplay triggering fall events in individuals.

### Conclusions

The results of the described study have the potential to expand knowledge regarding the complex interplay between environmental conditions, activities, and intrinsic fall risk factors triggering a fall event. The operationalization of the described prototypical dynamic fall risk model will enable the estimation of an individual fall risk and thereby improve several fields of fall prevention.

## Abbreviations

BW: body weight
FARSEEING: Fall Repository for the Design of Smart and Self-adaptive Environments Prolonging Independent Living
FRAT: Fall Risk Assessment Tool
MoS: margin of stability

## Data Availability

Part of the data will be presented in the main manuscript. Raw data are not publicly available to preserve individuals’ privacy under the European General Data Protection Regulation.
